# Prevalence, Host Range, and Comparative Genomic Analysis of Temperate *Ochrobactrum* Phages

**DOI:** 10.3389/fmicb.2017.01207

**Published:** 2017-06-30

**Authors:** Claudia Jäckel, Stefan Hertwig, Holger C. Scholz, Karsten Nöckler, Jochen Reetz, Jens A. Hammerl

**Affiliations:** ^1^Department of Biological Safety, German Federal Institute for Risk AssessmentBerlin, Germany; ^2^German Center for Infection Research, Bundeswehr Institute of MicrobiologyMunich, Germany

**Keywords:** *Ochrobactrum*, prophage, genome, temperate, lysogeny, phage, *Brucella*

## Abstract

*Ochrobactrum* and *Brucella* are closely related bacteria that populate different habitats and differ in their pathogenic properties. Only little is known about mobile genetic elements in these genera which might be important for survival and virulence. Previous studies on *Brucella* lysogeny indicated that active phages are rare in this genus. To gain insight into the presence and nature of prophages in *Ochrobactrum*, temperate phages were isolated from various species and characterized in detail. *In silico* analyses disclosed numerous prophages in published *Ochrobactrum* genomes. Induction experiments showed that *Ochrobactrum* prophages can be induced by various stress factors and that some strains released phage particles even under non-induced conditions. Sixty percent of lysates prepared from 125 strains revealed lytic activity. The host range and DNA similarities of 19 phages belonging to the families *Myoviridae, Siphoviridae*, or *Podoviridae* were determined suggesting that they are highly diverse. Some phages showed relationship to the temperate *Brucella inopinata* phage BiPB01. The genomic sequences of the myovirus POA1180 (41,655 bp) and podovirus POI1126 (60,065 bp) were analyzed. Phage POA1180 is very similar to a prophage recently identified in a *Brucella* strain isolated from an exotic frog. The POA1180 genome contains genes which may confer resistance to chromate and the ability to take up sulfate. Phage POI1126 is related to podoviruses of *Sinorhizobium meliloti* (PCB5), *Erwinia pyrifoliae* (Pep14), and *Burkholderia cenocepacia* (BcepIL02) and almost identical to an unnamed plasmid of the *Ochrobactrum intermedium* strain LMG 3301. Further experiments revealed that the POI1126 prophage indeed replicates as an extrachromosomal element. The data demonstrate for the first time that active prophages are common in *Ochrobactrum* and suggest that atypical brucellae also may be a reservoir for temperate phages.

## Introduction

*Ochrobactrum* species are non-fermenting, aerobic, gram-negative bacilli that are widespread in the environment and have been isolated from various ecological niches, such as water, soil, plants, and animals (Jelveh and Cunha, 1999; Möller et al., 1999; Lebuhn et al., 2000; Goris et al., 2003; Kämpfer et al., 2003; Bathe et al., 2006). Some *Ochrobactrum* strains belonging to different species were studied for their potential to degrade chemical pollutants and for heavy metal detoxification under a wide range of environmental conditions (El-Sayed et al., 2003; Zhang et al., 2006; Sultan and Hasnain, 2007). The α-proteobacterial genus *Ochrobactrum* belongs to the family *Brucellaceae* and contains to date 16 species, the type species of which is *Ochrobactrum anthropi* (Kämpfer et al., 2011). The closest relatives of *Ochrobactrum* spp. are brucellae (Scholz et al., 2008). *Ochrobactrum intermedium* e.g., shares 98.8% 16S rRNA gene similarity with *Brucella* spp. and is more closely related to this genus than to other *Ochrobactrum* species (Velasco et al., 1998; Lebuhn et al., 2000, 2006). However, while brucellae are well-recognized as important pathogens that cause brucellosis in man and many animal species, *Ochrobactrum* strains, particularly those belonging to the species *O. anthropi* and *O. intermedium*, have for a long time been regarded as opportunistic human pathogens of low virulence, infecting only immunocompromised patients with underlying diseases (Scholz et al., 2008). Nevertheless, the number of publications on opportunistic/nosocomial infections caused by *O. anthropi* has increased over the last decade (Shrishrimal, 2012; Mudshingkar et al., 2013; Siti Rohani and Tzar, 2013). Moreover, a rising number of reported cases included some potentially life-threatening infections, such as endocarditis (Mahmood et al., 2000; Daxboeck et al., 2002; Romero Gomez et al., 2004). The ability of *O. anthropi* to adhere to silicone may play a role in catheter-associated infections (Wi and Peck, 2010; Qasimyar et al., 2014). Some reports describe severe *O. anthropi* infections even in immunocompetent hosts with a clinical presentation similar to brucellosis (Kettaneh et al., 2003; Romero Gomez et al., 2004; Perez-Blanco et al., 2005; Ozdemir et al., 2006). *O. anthropi* often exhibits an intrinsic multi-resistance to antibiotics (Thoma et al., 2009; Johnning et al., 2013). While the location and transmission of virulence and antibiotic resistance genes is commonly associated with mobile genetic elements (MGE) like plasmids and phages, only little is known about MGE of *Ochrobactrum* and the related genus *Brucella*.

In *O. anthropi* ATCC 49188, four plasmids encoding several transporters have been identified which may contribute to the fitness of the strain (Chain et al., 2011). *Ochrobactrum* strain TD contained three linear plasmids, that allowed the strain to use vinyl chloride and ethene as growth substrates (Danko et al., 2004). By contrast, there are yet no reports on phages of *Ochrobactrum*. In *Brucella* the first temperate phage (BiPB01) has recently been described (Hammerl et al., 2016). BiPB01 revealed a broad host range within the genus *Brucella*. Its attachment site within the bacterial chromosome also exists in *Ochrobactrum*. Even though *Ochrobactrum* strains were not lysed by the phage, it showed significant DNA homologies to prophages of *Ochrobactrum*, particularly to a prophage residing in chromosome 1 of the *O. anthropi* strain ATCC 49188 indicating that temperate phages might occur in *Ochrobactrum* spp. as well.

In this study the presence of prophages in *Ochrobactrum* was determined followed by induction experiments to isolate and characterize temperate phages. *In silico* analyses revealed numerous prophages in the published sequences of 19 *Ochrobactrum* strains. Prophages could be induced by various stress factors. Phage particles were isolated from 19 lysates and characterized in terms of their morphology, host range and genetic relationship. The genomic sequences of two phages (POI1126 and POA1180) were determined and analyzed in detail.

## Materials and methods

### Bacterial strains, media, and growth conditions

All strains used in this study were obtained from the strain collections of the Bundeswehr Institute of Microbiology (Munich) and Federal Institute for Risk Assessment (Berlin). Information on the relevant strains is summarized in Table 1. If not otherwise indicated bacteria were cultivated aerobically in lysogeny broth (LB) (Carl Roth, Karlsruhe, Germany) at 28°C for 24 h under shaking conditions (180–200 rpm). Solid and overlay agar contained 1.8% (w/v) and 0.6% (w/v) bacto-agar No. 1 (Oxoid, Wesel, Germany), respectively.

**Table 1:** Bacterial strains used in this study.

***Ochrobactrum* spp. strains**	**Other designation, country (source)**	**Designation of the Bundeswehr Institute collection**
***O. anthropi***		
O1129	LMG3331, France (unknown)	B-1129
O1154	CCUG20020, Sweden (human blood)	B-1154
O1157	CCUG24695	B-1157
O1163	CCUG34461	B-1163
O1172	LMG3329	B-1172
O1178	LMG33, Denmark (human spinal fluid)	B-1178
O1180	WS4292	B-1180
O1199	DSM20150	B-1199
O1215	LMG5442	B-1215
***O. intermedium***		
O1126	LMG3301T, France (human blood)	LMG3301T
O1132	LMG3306, France (soil)	B-1132
O1135	OiC8-6	B-1135
O1147	CCUG1838	B-1147
O1191	TD30	B-1191
O1216	LMG5446, USA (bladder)	B-1216
O1218	CCM7036	B-1218
***O. tritici***		
O1114	LAIII-106	B-1114
O1184	WS1830	B-1184
O1187	WS1846	B-1187
***O. dulfi***		
O1205	CCUG30717, Sweden (human blood)	B-1205
O1206	CCUG43892, Norway (human, amniotic fluid)	B-1206
***O. pseudintermedium***		
O1164	CCUG34735, Sweden (water)	B-1164
***O. oryzae***		
O1196	DSM17471, India (deep water rice roots)	B-1196
***O. gallinifaeces***		
O1142	ISO1965	B-1142
***O. haemophilum***		
O1166	CCUG38531, Sweden (human blood)	B-1166

*ATCC, American Type Culture Collection; BCCM/LMG, Belgian Co-ordinated Collection of Microorganisms*.

### Prophage induction

To optimize the induction of *Ochrobactrum* prophages, various stress factors (mitomycin C, heat, UV) were investigated. At an adsorption (A_588nm_) of 0.2–0.3, 10 ml bacterial cultures were treated with different mitomycin C concentrations (0.5–10 μg/ml), heat (50, 60, or 70°C for 30 and 60 s) or UV (for 30 and 60 s). Thereafter, cultures were incubated under standard conditions for 16–18 h. UV treatment was performed by applying aliquots of the culture into petri dishes (*d* = 90 mm), which were placed in a distance of 10 cm to an UV lamp (corresponding 45 J m^−2^). UV radiation treatment was performed for 30 or 60 s. To obtain cell-free lysates, samples were centrifuged for 15 min at 6,000 × g and filtrated through 0.22 μm sterile filters (GE Healthcare, Munich, Germany). Lytic activity was quantified by spotting 10 μl aliquots of a 1:10 dilution series onto lawns of susceptible *Ochrobactrum* strains. All strains were investigated in triplicate under the respective induction conditions. For further characterization, plaques of POA1180 and POI1126 were purified by a three-fold single plaque purification procedure.

### Isolation, propagation, and purification of POA1180 and POI1126

Phages POA1180 and POI1126 were recovered by mitomycin C (0.5 μg/ml) treatment of strains *O. anthropi* O1180 and *O. intermedium* O1126, respectively. High titer lysates were prepared from 200 ml cultures. Cell-free lysates were obtained by centrifugation at 6,000 × g for 30 min and filtration of the supernatants through 0.22 μm sterile pore-size filters (GE Healthcare, Munich, Germany). To remove bacterial DNA and RNA, phage lysates were supplemented with 10 mM MgCl_2_, 10 μg ml^−1^ DNaseI and RNase A (Roche, Mannheim, Germany) followed by an incubation at 37°C for 2 h. Phage particles were concentrated by ultracentrifugation (Beckman) for 4 h at 200,000 × g. Concentrated phages were resuspended in SM-buffer and purified by discontinuous gradient (CsCl, 1.35–1.65 g ml^−1^) centrifugation at 141,000 × g for 18 h (Sambrook and Russell, 2001). After centrifugation phage bands were removed and desalted by 100K Amicon Ultra centrifugal filter columns (Merck Millipore, Schwalbach, Germany).

### Host range determination of the phages

The host range of the phages (e.g., POA1180, POI1126) was determined by spot assays. This was performed by applying 100–200 μl of each *Ochrobactrum* spp. strain to 6 ml LB soft agar (0.6%) and pouring of the overlay agar onto a LB agar plate. Aliquots of 1:10 serial dilutions of phage lysates were dropped onto the lawn of the solidified overlay agar. After 24 h spotting areas were visually inspected for plaque formation. Lytic activity was examined on strains of *Ochrobactrum* spp. (*n* = 119), *Brucella* spp. (26 reference and type strains), *Mesorhizobium* (*n* = 6), *Sinorhizobium* (*n* = 5), *Pseudomonas* (*n* = 5) and *Yersinia enterocolitica* O:9 (*n* = 4).

### Transmission electron microscopy

CsCl-purified phages were applied to pioloform-carbon-coated, 400-mesh copper grids (Plano GmbH, Germany), for 10 min, fixed with 2.5% aqueous glutaraldehyde solution for 1 min, stained with 2% aqueous uranyl acetate solution for 1 min and examined using a JEM-1010 (JOEL, Tokyo, Japan) transmission electron microscope at 80 kV accelerated voltage.

### Extraction of phage DNA and sequencing

Phage DNA was extracted from CsCl-purified particles by proteinase K/SDS treatment and ethanol precipitation (Sambrook and Russell, 2001). Thereafter, the phage DNA was resuspended in 0.5 × TE-buffer (pH 8.0) for further analyses. Sequencing libraries were prepared using the Nextera XT DNA Sample Preparation Kit according to the recommendations of the manufacturer. Paired-end sequencing was performed on the Illumina MiSeq benchtop using the MiSeq Reagent v3 600-cycle Kit (2 × 300 cycles) (Illumina, CA, USA). Raw reads were assembled *de novo* using tools of the Pathosystems Resource Integration Center resulting in a single contig with an average sequence coverage of more than 120 and 100 per consensus base for POA1180 and POI1126, respectively. DNA hybridization was conducted using the Roche digoxygenin hybridization system on positively charged nylon membranes according to the manufacture's procedure (Roche, Heidelberg, Germany).

### Bioinformatic analysis

To identify putative prophage sequences in the available *Ochrobactrum* spp. genomes of GenBank (NCBI), the Phage Search Tool-PHAST was used (Zhou et al., 2011). Sequence analysis and alignments were carried out using Accelrys Gene v2.5 (Accelrys Inc., San Diego, CA, USA). Identification of genetic elements like ORFS, transcription terminators, and tRNAs on the phage genome was conducted as previously described (Hammerl et al., 2011, 2016). Similarity and identity values were determined at the NCBI homepage using different BLAST algorithms (Johnson et al., 2008). Annotation of the phage genomes was performed by using Sequin (https://www.ncbi.nlm.nih.gov/Sequin/).

### Nucleotide sequence accession numbers

The complete nucleotide sequences of the *O. anthropi* POA1180 and *O. intermedium* POI1126 phage genomes were submitted to GenBank under the accession numbers KX669658 and KY417925, respectively.

## Results

### Prophage sequences are widely distributed in *Ochrobactrum*

To get a first overview on the presence of prophage DNA in *Ochrobactrum* spp., 19 strains representing several species whose genome sequences have been published, were analyzed by automated *in silico* analyses using the PHAge Search Tool (PHAST) (Zhou et al., 2011). We identified prophage sequences (6.9–91.8 kb in size) in all analyzed *Ochrobactrum* species. Between two and ten putative phage genomes were detected in each strain (Table 2). Highly prevalent was a 17.3–91.8 kb DNA region that showed significant similarity to the *Sinorhizobium meliloti* phage 16-3, which possesses a genome of 60 kb (Deak et al., 2010). The second most frequent prophage was represented by a DNA region of 25.3–56.8 kb revealing close relationship to phage AmM-1 isolated from the Rhizobiales deep-sea bacterium *Aurantimonas* sp. whose genome has a size of 47.8 kb (Yoshida et al., 2015). Most of the remaining prophages in *Ochrobactrum* spp. are similar to other *Rhizobium* phages but homologies to phages of *Rhodobacter, Pseudomonas*, various *Enterobacteriaceae* and even *Bacillus* were also detected (Table 2). According to the results obtained by *in silico* analyses, some of the prophages may be complete and intact. The GC content of the prophages ranges between 49.7 and 65% whereas the average GC content of *O. anthropi* is 56%, suggesting that some *Ochrobactrum* strains may have acquired temperate phages from different hosts through horizontal gene transfer (Table S2).

**Table 2:** Prophage content of published *Ochrobactrum* sp. genomes.

**Strain**	**Intact (Size)**	**Questionable (Size)**	**Incomplete (Size)**
***O. anthropi***			
ATCC 49188	3 (17.2–22.3 kb)	n.d.	2 (17.5–22.6 kb)
ATCC 49687 (OAB)	n.d.	1 (18.0 kb)	1 (13.0 kb)
ML7	3 (41.0–56.8 kb)	3 (12.5–17.8 kb)	n.d.
W13P3	n.d.	5 (15.3–46.3 kb)	1 (7.1 kb)
60a	n.d.	n.d.	2 (8.2–10.5 kb)
CTS-325	n.d.	2 (11.7–22.7 kb)	n.d.
FRAF13	1 (25.3 kb)	1 (54.0 kb)	2 (8.5–26.1 kb)
***O. intermedium***			
LMG 3301	2 (40.8–48.8 kb)	1 (33.1 kb)	1 (27.4 kb)
2745-2	1 (51.0 kb)	3 (15.5–40.1 kb)	n.d.
M86	3 (23.5–54.3 kb)	4 (9.2–30.5 kb)	2 (6.9–17.3 kb)
CCUG 57381 (299E)	n.d.	n.d.	2 (8.3–10.6 kb)
2745-2	1 (51.0 kb)	n.d.	n.d.
KCJK1738	1 (25.3 kb)	1 (54.0 kb)	2 (8.5–26.1 kb)
***Ochrobactrum* spp**.			
*O. pseudogrignonense* K8	2 (13.1–13.7 kb)	1 (36.9 kb)	n.d.
*Ochrobactrum* sp. CDB2	2 (13.8–61.9 kb)	n.d.	1 (26.4 kb)
*O. rhizosphaerae* SJY1	6 (13.9–91.8 kb)	n.d.	1 (15.1 kb)
*Ochrobactrum* sp. EGD-AQ16	n.d.	n.d.	3 (7.5–14.9 kb)
*Ochrobactrum* sp. UNC390CL2Tsu3S39 BS36	1 (23.1 kb)	n.d.	2 (10.2–26.5 kb)

*n.d., Not detected*.

### *Ochrobactrum* prophages can be easily induced by stress factors

**Figure 1:**
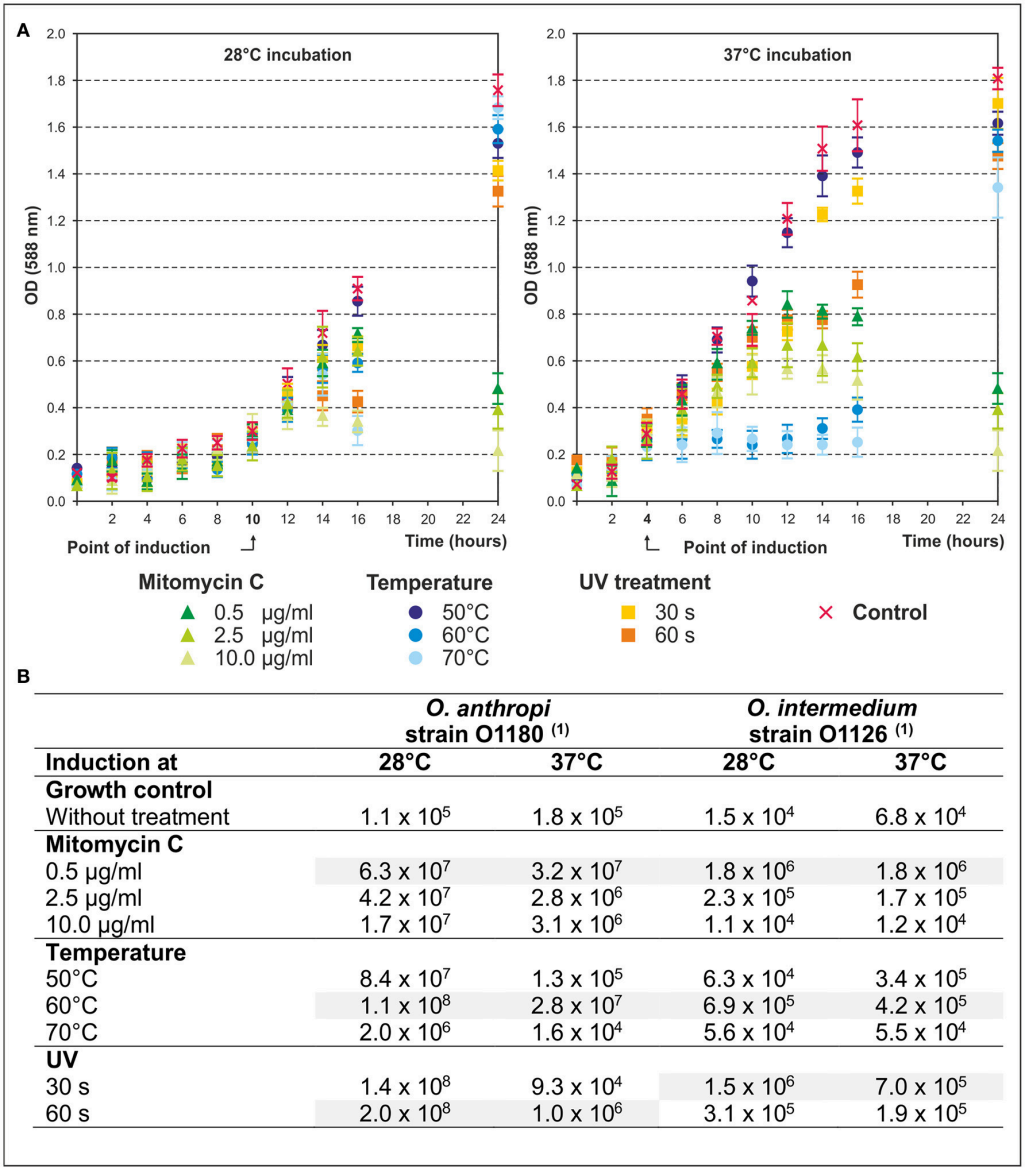
Efficacy of phage release during mitomycin C, temperature, and UV treatment. **(A)** In the diagrams the bacterial growth of *O. anthropi* strain O1180 during stress treatment at 28 and 37°C is shown. Given are mean values of three independent experiments. Error bars indicate the standard deviation from the mean value. The point of induction (OD_588nm_) is indicated. **(B)** The table summarizes the results of the phage induction by different stress factors. The phage titer is given in pfu/ml. (1) Plaque formation was investigated on *O. anthropi* strain O1129.

In this study, 125 *Ochrobactrum* strains of various species deposited in the strain collection of the BfR were analyzed in terms of inducible prophages. We first compared the efficacy of various stress factors (mitomycin C, UV, high temperature) on the release of phage particles. For this purpose, the strains *O. anthropi* O1180 (Figure 1A) and *O. intermedium* O1126 (data not shown) were treated with different concentrations (0.5, 2.5, and 10.0 μg/ml) of mitomycin C, heat (50, 60, 70°C for 30 and 60 s) and UV (45 J m^−2^ for 30 and 60 s). Thereafter, the strains were further incubated at 28 and 37°C. At both temperatures bacterial growth was inhibited in response to the tested stress factors, particularly mitomycin C, even though prophage induction at 37°C occurred earlier in most preparations. As shown in Figure 1B even the untreated control of both strains released 10^4^ to 10^5^ phages per milliliter (Table 3). Under stress conditions titers up to 2 × 10^8^ pfu/ml were determined. In general, all tested stress factors were suitable to induce prophages, albeit phage titers achieved in the individual approaches differed significantly from each other. *O. anthropi* strain O1180 revealed much stronger temperature dependence than *O. intermedium* O1126. Some phage titers obtained at 28°C were two to three orders of magnitude higher than those of the corresponding lysates at 37°C. Moreover, whereas UV treatment was the most efficient method to induce prophages in *O. anthropi*, the highest phage titers in *O. intermedium* were achieved with a mitomycin C concentration of 0.5 μg/ml. Thus, *Ochrobactrum* strains diverge significantly regarding their sensitivity to prophage inductors. Based on the results of this study, subsequent induction experiments with a broad range (*n* = 125) of *Ochrobactrum* spp. strains were performed with 0.5 μg/ml mitomycin C at 28°C.

**Table 3:** Spontaneous lysis of *Ochrobactrum* spp. strains.

***Ochrobactrum* spp. strain**	**Number of released phages**	**Host strain**
***O. anthropi***		
O1178	4 × 10^4^ pfu/ml	*O. intermedium* O1218
O1129	–	*O. intermedium* O1147
O1157	–	*O. intermedium* O1147
O1154	–	*O. intermedium* O1147
O1180	1 × 10^5^ pfu/ml	*O. anthropi* O1129
***O. intermedium***		
O1216	2 × 10^4^ pfu/ml	*O. anthropi* O1199
O1218	–	*O. intermedium* O1135
O1132	–	*O. anthropi* O1163
O1191	–	*O. anthropi* O1215
O1126	2 × 10^4^ pfu/ml	*O. anthropi* O1129
***O. tritici***		
O1114	2 × 10^5^ pfu/ml	*O. anthropi* O1172
O1187	2 × 10^5^ pfu/ml	*O. anthropi* O1199
***O. dulfi***		
O1206	–	*O. anthropi* O1199
O1205	–	*O. anthropi* O1215
***O. pseudintermedium***		
O1164	–	*O. intermedium* O1135

The 125 prepared lysates were tested for their lytic activity by spotting aliquots on all 125 *Ochrobactrum* spp. strains. As an additional indicator for released particles, DNA was isolated from the lysates. Plaques were observed with ~60% of the lysates. Up to 20 strains were susceptible to each lysate. In most cases several *Ochrobactrum* species were lysed. To get a deeper insight into the released phages, 19 lysates originating from *O. anthropi, O. intermedium, Ochrobactrum tritici, O. dulfi*, and *Ochrobactrum* sp., from which significant amounts of encapsidated phage DNA could be isolated (data not shown) were selected for further analyses. Electron microscopic examination revealed phage particles in all lysates, even though four of them did not show lytic activity. According to their morphology, the identified phages belong to different families (Table 4). Most prominent was the family *Myoviridae* represented by phages with an isodiametric or elongated head and a contractile tail. Some lysates contained particles with a long, non-contractile and very short tail, which are members of the *Siphoviridae* and *Podoviridae*, respectively. However, it is important to mention that a number of lysates exhibited various morphotypes indicating that the respective strains released several phages simultaneously.

**Table 4:** Morphology of *Ochrobactrum* spp. phages.

**Phage**	**Family**
***O. anthropi***	
P1178	*Myoviridae*
P1157	*Siphoviridae, icosahedral*
P1154	n.d.
P1129	*Podoviridae*
POA1180	*Myoviridae*
***O. intermedium***	
P1216	*Myoviridae*
P1218	*Siphoviridae, icosahedral*
P1191	*Siphoviridae, icosahedral*
P1132	*Myoviridae*
POI1126	*Podoviridae*
***O. tritici***	
P1114	*Myoviridae*
P1184	*Myoviridae*
P1187	*Podoviridae*
***O. dulfi***	
P1206	*Myoviridae*
P1205	*Siphoviridae, icosahedral*
***Ochrobactrum* spp**.	
P1164	*Myoviridae*
P1142	*Siphoviridae, prolate*
P1196	*Myoviridae*
P1166	*Siphoviridae*

n.d., Not determined

### Host range and genetic relationships of the isolated *Ochrobactrum* phages

**Figure 2:**
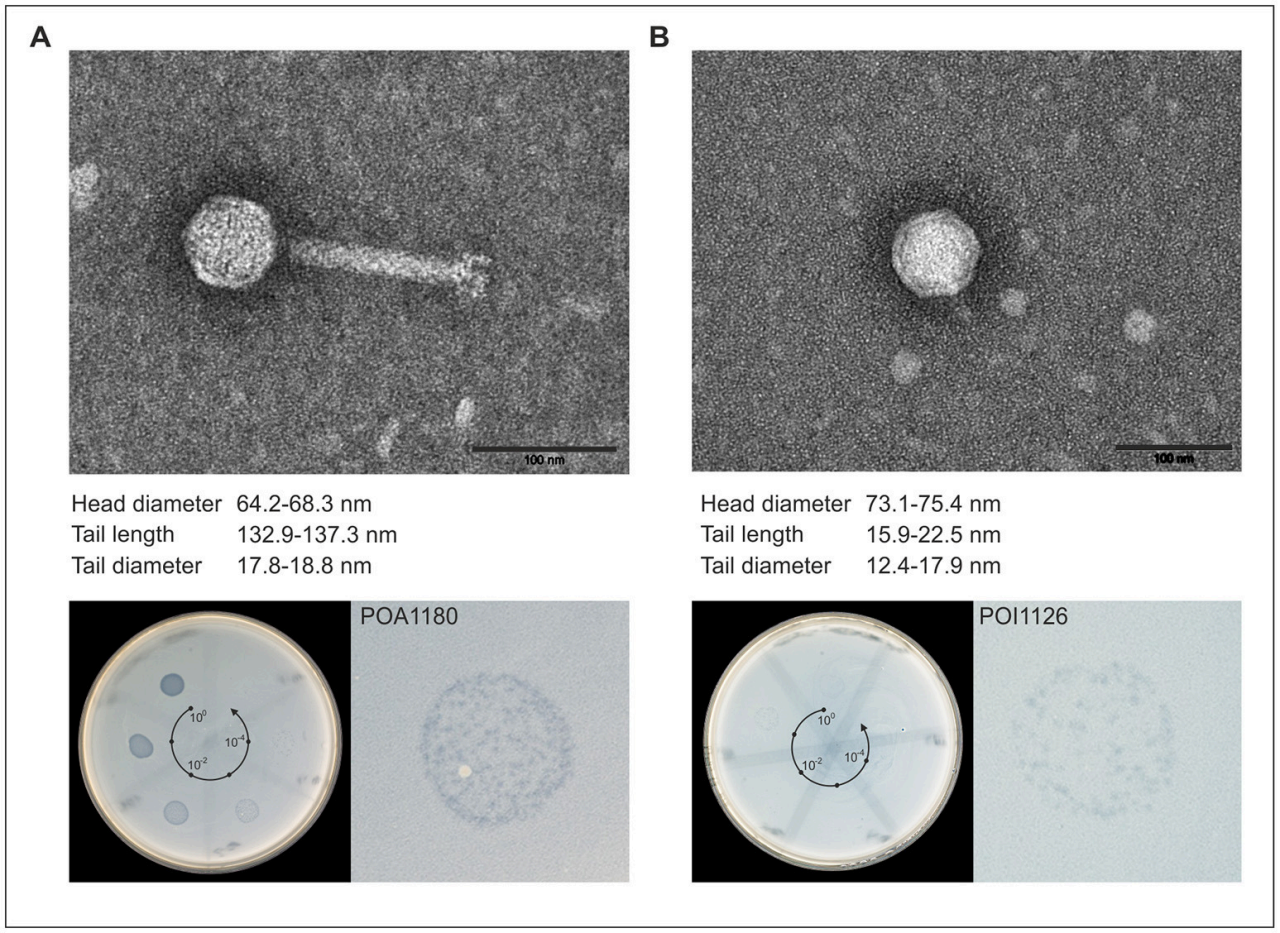
Morphology and lytic activity of POA1180 and POI1126. In the upper part, transmission electron micrographs (TEM) of POA1180 **(A)** and POI1126 **(B)** phage particles isolated from *Ochrobactrum* strains O1180 and O1126, respectively, by mitomycin C induction are shown. The black bar represents a size standard of 100 nm. The lower part demonstrates the lytic activity of the phages POA1180 **(A)** and POI1126 **(B)**. The left panels show the lytic activity of the indicated phages during spot testing. On the bacterial lawn of *O. anthropi* strain O1129, 10 μl of a 1:10 dilution series (spot 1–6) of the P1180 and P1126 phage lysates was applied. The right panels show a six-fold magnification of a representative spotting zone comprising single plaques.

Previous experiments demonstrated that most of the investigated *Ochrobactrum* strains harbored one or more prophages inducible by mitomycin C, heat and UV. To obtain data on individual phages present in the 19 selected lysates, high titer lysates were prepared by mitomycin C treatment of large culture volumes of the respective *Ochrobactrum* strains (see Section Materials and Methods). Upon purification by CsCl density-gradient centrifugation, 19 phages were isolated and characterized in terms of their host range and genetic relationship. Similar to the data obtained with non-purified lysates, the majority of phages infected several *Ochrobactrum* species, particularly *O. anthropi* and *O. intermedium*, but also strains of *O. tritici, O. oryzae*, and *O. pseudintermedium* (Table 5). By contrast, classical and atypical *Brucella* strains were not lysed. Plaques were even and mostly small and rather turbid, as shown for the myovirus POA1180 and the podovirus POI1126 (Figure 2). On the other hand, phage P1218 isolated from *O. intermedium* revealed a very narrow host range and exclusively lysed three strains belonging to this species. Finally, four phages (P1142, P1166, P1184 and P1196) did not infect any *Ochrobactrum* spp. strain. In some cases growth inhibition of indicator strains was observed indicating that phage replication did not occur in those strains.

**Table 5:** Lytic activity of the purified phages.

	***O. anthropi***					***O. intermedium***					***O. tritici***			***O. dulfi***		***Ochrobactrum* spp**.			
	**POA1180**	**P1178**	**P1129**	**P1157**	**P1154**	**P1216**	**P1218**	**POI1126**	**P1132**	**P1191**	**P1114**	**P1187**	**P1184**	**P1206**	**P1205**	**P1164**	**P1196**	**P1142**	**P1166**
***O. anthropi* strains (*n* = 64)**																			
O1180	−	−	−	−	−	−	−	−	−	−	−	−	−	−	−	−	−	−	−
O1145	−	−	−	−	−	−	−	−	−	−	−	−	−	−	−	−	−	−	−
O1124	−	−	−	−	−	−	−	−	−	−	−	−	−	−	−	−	−	−	−
O1162	−	−	−	−	−	−	−	−	−	−	−	−	−	−	−	−	−	−	−
O1148	−	−	−	−	−	−	−	−	−	−	−	−	−	−	−	−	−	−	−
O1122	+	−	+	−	–	−	−	+	−	−	−	+	−	−	−	−	−	−	−
O1120	−	−	+	−	−	+	−	+	−	−	−	+	−	−	−	−	−	−	−
O1172	−	−	−	−	−	−	−	+	−	−	+	−	−	−	−	−	−	−	−
O1119	−	−	−	−	−	−	−	+	−	−	−	−	−	−	−	−	−	−	−
O1178	−	−	−	−	−	+	−	+	−	−	−	+	−	−	−	−	−	−	−
O1174	−	−	−	−	−	+	−	+	−	−	−	+	−	−	−	−	−	−	−
O1200	−	−	−	−	−	−	−	−	−	−	−	−	−	−	−	−	−	−	−
O1151	−	−	−	−	−	−	−	+	−	−	−	−	−	−	−	−	−	−	−
O1181	+	−	−	−	−	−	−	−	−	−	−	−	−	−	−	−	−	−	−
O1222	−	−	−	−	−	−	−	−	−	−	−	+	−	−	−	−	−	−	−
O1153	−	−	−	−	−	−	−	−	−	−	−	−	−	−	−	−	−	−	−
O1171	−	−	−	−	−	−	−	−	−	−	−	−	−	−	−	−	−	−	−
O1150	−	−	−	−	−	−	−	−	−	−	−	−	−	−	−	−	−	−	−
O1173	+	−	+	−	−	−	−	+	−	−	−	+	−	−	−	−	−	−	−
O1177	−	−	−	−	−	−	−	−	−	−	−	−	−	−	−	−	−	−	−
O1152	−	−	−	−	−	−	−	+	−	−	−	−	−	−	−	−	−	−	−
O1160	−	−	−	−	−	−	−	−	−	−	−	−	−	−	−	−	−	−	−
O1223	−	−	+	−	−	+	−	+	−	−	(+)	+	−	−	−	−	−	−	−
O1116	+	−	+	−	−	−	−	+	−	−	−	+	−	−	−	−	−	−	−
O1130	−	−	+	−	−	−	−	+	−	−	−	−	−	−	−	−	−	−	−
O1159	−	−	−	−	−	−	−	−	−	−	−	−	−	−	−	−	−	−	−
O1163	−	−	−	−	−	−	−	−	+	−	−	−	−	−	−	−	−	−	−
O1158	−	−	−	−	−	−	−	−	−	−	−	−	−	−	−	−	−	−	−
O1156	+	−	−	−	(+)	−	−	+	−	(+)	−	−	−	−	−	−	−	−	−
O1125	−	−	+	−	−	−	−	+	−	−	−	−	−	−	−	−	−	−	−
O1212	−	−	−	−	−	−	−	−	−	−	−	−	−	−	−	−	−	−	−
O1143	−	−	−	−	−	−	−	+	−	−	−	−	−	−	−	−	−	−	−
O1161	−	−	−	−	−	−	−	−	−	−	−	−	−	−	−	−	−	−	−
O1149	−	+	−	−	−	−	−	+	−	−	−	−	−	−	−	−	−	−	−
O1169	−	−	−	−	−	−	−	−	−	−	−	−	−	−	−	−	−	−	−
O1144	−	−	−	−	−	−	−	−	−	−	−	−	−	−	−	−	−	−	−
O1115	−	−	−	−	−	−	−	−	−	−	−	−	−	−	−	−	−	−	−
O1199	−	−	−	−	−	+	−	−	−	−	−	−	−	−	−	−	−	−	−
O1207	−	−	−	−	−	−	−	+	−	−	−	+	−	−	−	−	−	−	−
O1211	−	−	−	−	−	−	−	−	−	−	−	−	−	−	−	−	−	−	−
O1213	−	−	−	−	−	−	−	−	−	−	−	−	−	−	−	−	−	−	−
O1224	+	−	+	−	−	−	−	+	−	−	−	−	−	−	−	−	−	−	−
O1227	+	−	+	−	−	−	−	+	−	−	−	−	−	−	−	−	−	−	−
O1217	−	−	−	−	−	−	−	−	−	−	−	−	−	−	−	−	−	−	−
O1209	+	−	−	−	−	−	−	−	−	−	−	−	−	−	−	−	−	−	−
O1201	−	−	−	−	−	−	−	+	−	−	−	−	−	−	−	−	−	−	−
O1215	−	−	−	−	−	−	−	−		+	−	−	−	−	+	−	−	−	−
O1210	−	−	−	−	−	−	−	−	−	−	−	−	−	−	−	−	−	−	−
O1197	−	−	−	−	−	−	−	−	−	−	−	−	−	−	−	−	−	−	−
O1214	−	−	−	−	−	−	−	+	−	−	−	−	−	−	−	−	−	−	−
O1225	−	−	−	−	−	−	−	−	−	−	−	−	−	−	−	−	−	−	−
O1121	−	−	−	−	−	−	−	−	−	(+)	−	−	−	−	−	−	−	−	−
O1165	−	−	−	−	−	−	−	−	−	−	−	−	−	−	−	−	−	−	−
O1155	+	+	−	+	(+)	−	−	+	−	−	−	−	−	−	(+)	−	−	−	−
O1175	+	−	−	−	−	−	−	−	−	(+)		−	−	−	−		−	−	−
O1226	−	−	−	−	−	−	−	−	−	−	−	−	−	−	−	−	−	−	−
O1179	−	(+)	−	+	−	−	−	+	−	−	−	−	−	−	−	+	−	−	−
O1123	−	−	−	−	−	−	−	−	−	−	−	−	−	−	−	−	−	−	−
O1176	+	−	−	−	−	−	−	+	−	−	−	−	−	−	−	−	−	−	−
O1202	−	−	−	−	−	−	−	−	−	−	−	−	−	−	−	−	−	−	−
O1129	+	−	−	−	−	+	−	−	−	−	−	+	−	−	−	−	−	−	−
O1157	−	−	−	−	−	−	−	+	−	−	−	−	−	−	−	−	−	−	−
O1154	+	−	−	+	−	−	−	−	−	−	−	−	−	−	−	−	−	−	−
O1128	−	−	−	−	−	−	−	−	−	−	−	−	−	−	−	−	−	−	−
***O. intermedium* strains (*n* = 18)**																			
O1192	−	−	−	−	−	−	−	−	−	−	−	−	−	−	−	−	−	−	−
O1167	−	−	−	−	−	−	−	−	−	−	−	−	−	−	−	−	−	−	−
O1194	−	−	+	−	−	−	−	+	−	−	−	+	−	−	−	−	−	−	−
O1193	−	+	−	−	−	+	−	−	−	−	−	−	−	−	−	−	−	−	−
O1183	−	−	−	−	−	+	−	+	−	−	−	−	−	−	−	−	−	−	−
O1190	+	−	−	−	−	−	−	−	−	−	−	−	−	−	−	−	−	−	−
O1168	−	−	−	+	−	+	(+)	+	−	−	−	+	−	−	−	−	−	−	−
O1133	−	−	−	−	−	−	−	−	−	−	−	−	−	−	−	−	−	−	−
O1216	−	−	−	−	−	−	−	−	−	−	−	−	−	−	+	−	−	−	−
O1134	−	−	−	−	−	−	−	−	−	−	−	−	−	−	−	−	−	−	−
O1135	−	−	−	−	−	−	+	−	−	−	−	−	−	−	−	−	−	−	−
O1182	−	−	−	−	−	−	−	−	−	(+)	−	+	−	+	−	−	−	−	−
O1132	−	−	−	−	−	−	(+)	−	−	(+)	−	−	−	−	−	−	−	−	−
O1220	−	(+)	+	+	−	−	−	+	−	−	−	+	−	−	−	+	−	−	−
O1147	−	−	+	+	+	+	−	+	+	(+)	−	+	−	+	(+)	+	−	−	−
O1126	−	−	−	−	−	−	−	−	−	−	−	+	−	−	−	+	−	−	−
O1218	−	+	−	−	−	−	−	+	−	−	−	+	−	−	−	−	−	−	−
O1191	−	−	−	−	−	−	−	−	−	(+)	−	−	−	−	−	−	−	−	−
***O. tritici* strains (*n* = 9)**																			
O1221	−	−	−	−	−	−	−	−	−	−	−	−	−	−	−	−	−	−	−
O1114	−	−	−	−	−	−	−	−	−	−	−	−	−	−	−	−	−	−	−
O1187	−	−	−	−	−	−	−	−	−	−	−	−	−	−	−	−	−	−	−
O1184	−	−	−	−	−	−	−	−	−	−	−	−	−	−	−	−	−	−	−
O1170	−	−	−	−	−	−	−	−	−	−	−	−	−	−	−	−	−	−	−
O1208	−	−	−	−	−	−	−	−	−	−	−	−	−	−	−	−	−	−	−
O1195	−	(+)	−	+	−	−	−	+	−	−	−	−	−	−	−	+	−	−	−
O1140	−	−	−	−	−	−	−	−	−	−	−	−	−	−	−	−	−	−	−
O1146	−	−	−	−	−	−	−	−	−	−	−	−	−	−	−	−	−	−	−
***O. dulfi* strains (*n* = 3)**																			
O1206	−	−	−	−	−	−	−	−	−	−	−	−	−	−	−	−	−	−	−
O1205	−	−	−	−	−	−	−	−	−	−	−	−	−	−	−	−	−	−	−
O1198	−	−	−	−	−	−	−	(+)	−	−	−	−	−	−	−	+	−	−	−
***O. pseudogrignonense* strains (*n* = 3)**																			
O1219	−	−	−	−	−	−	−	−	−	−	−	−	−	−	−	−	−	−	−
O1203	−	−	−	−	−	−	−	−	−	−	−	−	−	−	−	−	−	−	−
O1189	+	−	−	−	−	−	−	−	−	−	−	−	−	−	−	−	−	−	−
***O. gallinifaecis* strains (*n* = 2)**																			
O1141	−	−	−	−	−	−	−	−	−	−	−	−	−	−	−	−	−	−	−
O1142	−	−	−	−	−	−	−	−	−	−	−	−	−	−	−	−	−	−	−
***O. grignonense* strains (*n* = 2)**																			
O1118	−	−	−	−	−	−	−	−	−	−	−	−	−	−	−	−	−	−	−
O1117	−	−	−	−	−	−	−	−	−	−	−	−	−	−	−	−	−	−	−
***O. lupini* strains (*n* = 2)**																			
O1136	−	−	−	−	−	−	−	−	−	−	−	−	−	−	−	−	−	−	−
O1137	−	−	−	−	−	−	−	−	−	−	−	−	−	−	−	−	−	−	−
***O. pseudintermedium* strains (*n* = 2)**																			
O1164	−	−	−	−	−	−	−	−	−	−	−	−	−	−	−	−	−	−	−
O1204	−	+	−	+	−	−	−	−	−	(+)	−	−	−	−	+	−	−	−	−
***O. haemophilum* strains (*n* = 1)**																			
O1166	−	−	−	−	−	−	−	−	−	−	−	−	−	−	−	−	−	−	−
***O. oryzae* strains (*n* = 1)**																			
O1196	−	−	+	−	−	+	−	+	−	−	−	+	−	+	−	+	−	−	−
***Mesorhizobium* sp. strains (*n* = 2)**																			
O1188	−	−	−	−	−	−	−	−	−	−	−	−	−	−	−	−	−	−	−
O1186	−	−	−	−	−	−	−	−	−	−	−	−	−	−	−	−	−	−	−
***Pseudomonas kiredjianae* strains (*n* = 1)**																			
O1139	−	−	−	−	−	−	−	−	−	−	−	−	−	−	−	−	−	−	−
***Pseudomonas saccharolyticum* strains (*n* = 2)**																			
O1138	−	−	−	−	−	−	−	−	−	−	−	−	−	−	−	−	−	−	−
O1127	−	−	−	−	−	−	−	−	−	−	−	−	−	−	−	−	−	−	−

*+, Strong phage activity; (+), weak phage activity; −, no phage activity*.

*The gray values indicate a phage activity*.

**Figure 3:**
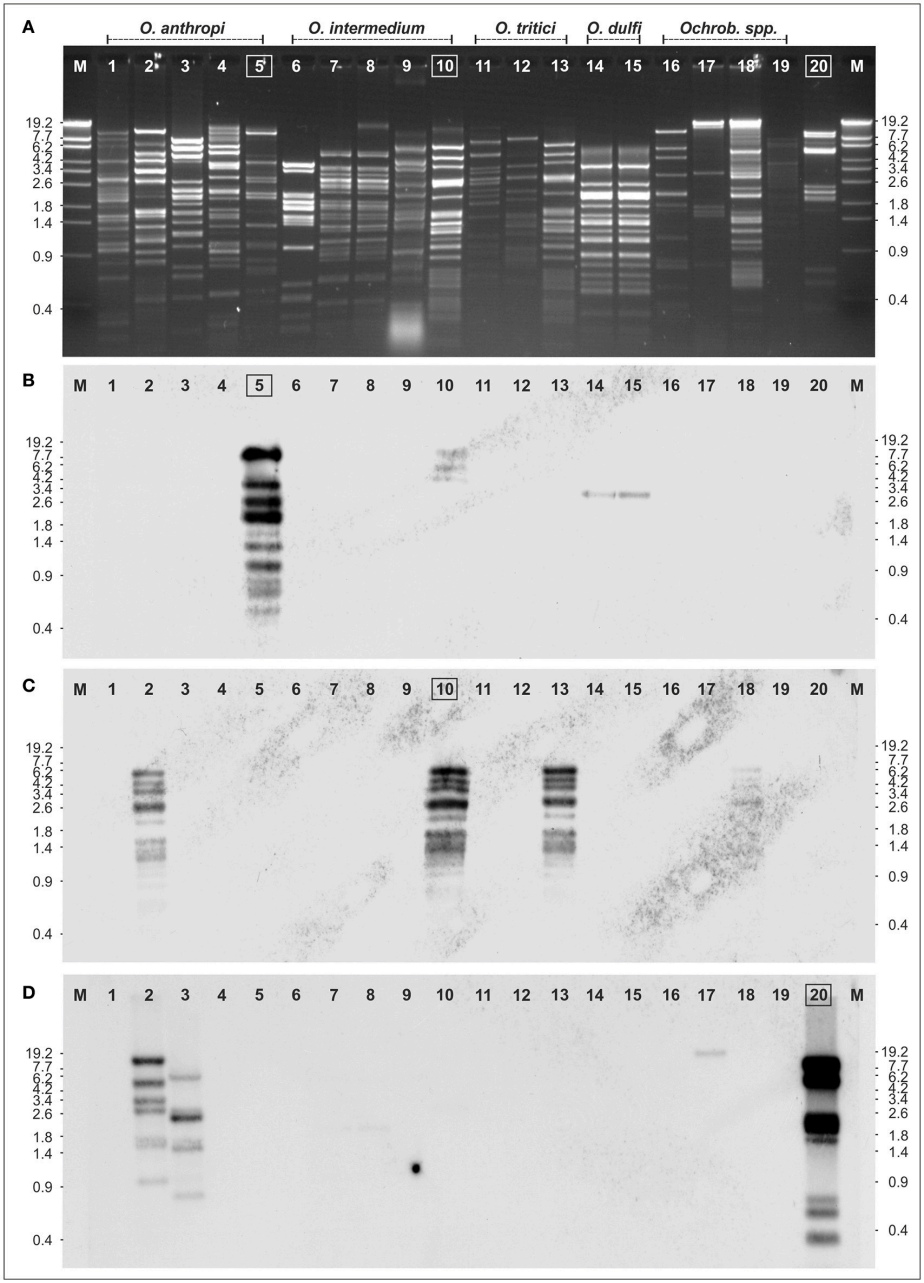
Relationship of 19 *Ochrobactrum* spp. phages. **(A)** EcoRV restriction patterns of the phages. Southern hybridization of POA1180 **(B)**, POI1126 **(C)**, and BiPBO1 **(D)** to the isolated *Ochrobactrum* spp. phage DNAs: *Ochrobactrum anthropi* phages P1178 (lane 1), P1129 (lane 2), P1157 (lane 3), P1154 (lane 4), POA1180 (lane 5), *O. intermedium* phages: P1216 (lane 6), P1135 (lane 7), P1132 (lane 8), P1218 (lane 9), POA1126 (lane 10), *O. tritici* phages: P1114 (lane 11), P1184 (lane 12), P1187 (lane 13), *O. dulfi* phages: P1206 (lane 14), P1205 (lane 15), *Ochrobactrum* spp. phages: P1164 (lane 16), P1142 (lane 17), P1166 (lane 18), P1189 (lane 19), and *Brucella inopinata* phage BiPB01 (lane 20). Lanes M, Lambda Eco130I marker DNA.

To determine the genetic relationships of the *Ochrobactrum* phages amongst themselves and to the temperate *Brucella inopinata* phage BiPBO1, restriction patterns of the phage genomes were compared, followed by Southern hybridization (Figure 3). EcoRV restriction analyses revealed numerous fragments and demonstrated that the phages possess double-stranded DNA. Most restriction patterns were unique but the *O. dulfi* phages P1205 and P1206 exhibited identical profiles. In addition, two *O. intermedium* phages (P1132 and P1135) and the phages P1126 and P1187, isolated from *O. intermedium* and *O. tritici*, respectively, showed very similar patterns. However, the latter two phages differ in their host specificity indicating at least some minor genetic variations. According to the obtained restriction fragments (Figure 3A), genome sizes between 32 and 63 kb were calculated (data not shown). To determine DNA homologies between the phages, Southern hybridizations were performed using three phage DNAs as probes; myovirus POA1180 isolated from *O. anthropi* (Figure 3B), podovirus POI1126 (Figure 3C) isolated from *O. intermedium* and *B. inopinata* phage BiPBO1 (Figure 3D), which already revealed significant homologies to some *Ochrobactrum* prophages (Hammerl et al., 2016). Phage POA1180 did not hybridize to most of the other phages. Weak homologies were detected to POI1126 and to the two *O. dulfi* phages whose restriction patterns were identical. Phage POI1126 hybridized strongly to the *O. anthropi* phage P1129 and to the *O. tritici* phage P1187. Moreover, the homologous restriction fragments were similar in size corroborating the close relationship of these phages. The temperate *B. inopinata* phage BiPBO1 showed relationship to the *O. anthropi* phages P1129 and P1157 (Figure 3). Based on these results it can be suggested that temperate *Ochrobactrum* phages are genetically highly diverse. To further characterize this diversity, the genomic sequences of myovirus POA1180 and podovirus POI1126 were determined.

**Figure 4:**
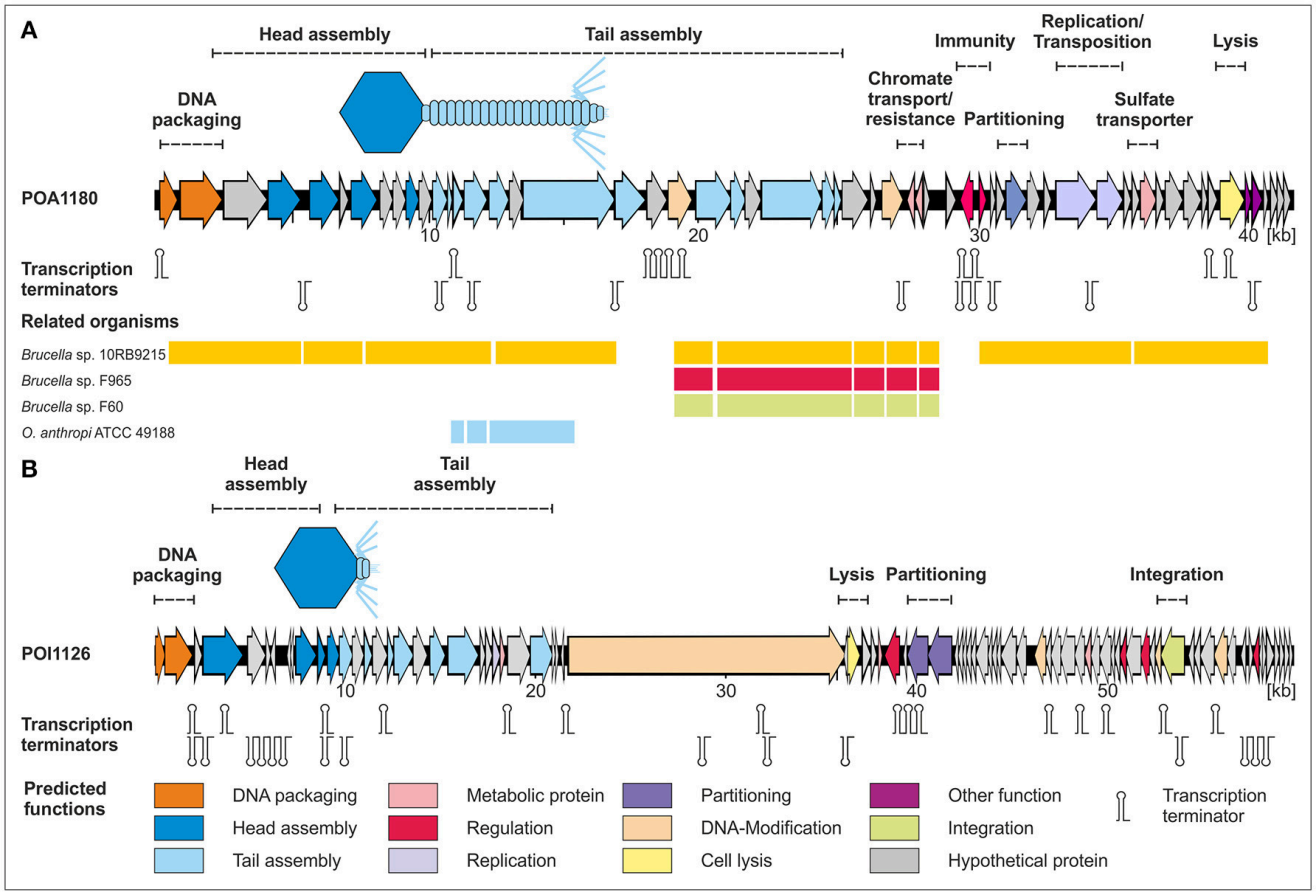
Gene maps of the *Ochrobactrum* phages POA1180 **(A)** and POI1126 **(B)**. Putative genes are colored according to the predicted functions of their products. The position of putative Rho-independent transcription terminators and tRNAs are indicated. For POA1180 nucleotide similarities (>75%) to closely related *Brucella* and *Ochrobactrum* genomes are shown.

The genome of phage POA1180 has a size of 41,655 bp with a GC-content of 56.6%. Fifty-eight putative gene products were assigned of which 55 are located on the same DNA strand. For 32 gene products a functional prediction could be made (Table S1). It is notable that almost no similarities were detected to proteins of other phages. Instead, very similar proteins are encoded by other *Ochrobactrum* strains and strains of the genera *Brucella, Agrobacterium, Bartonella, Burkholderia, Rhizobium*, and *Stappia*. The highest similarities exist to a prophage recently identified in *Brucella* strain 10RB9215 isolated from an exotic frog (Scholz et al., 2016). This indicates that closely related prophages are widespread in these bacteria but that reports on the respective phages are scarce. Based on homologies to other proteins, a gene map of phage POA1180 was constructed (Figure 4). The left half of the POA1180 genome mainly contains genes for DNA packaging and virion assembly. We identified putative genes for the small (ORF01) and large subunit of the terminase (ORF02), capsid protein, and several tail proteins (e.g., tail tube protein, tape measure protein, and tail fiber protein). The right half of the POA1180 genome contains genes for various proteins. First and foremost, two genes (ORF31 and ORF32) were identified which may confer resistance to chromium. Their products are very similar to proteins of *Rhizobium, Mesorhizobium*, and *Sinorhizobium*. While ORF31 encodes a chromate transport protein, the ORF32 product is closely related to chromate resistance proteins. Chromium resistance has already been reported for the *O. tritici* strain 5bvl1 but in this strain, the resistance genes are located on a transposon (Morais et al., 2011). Transposase genes (ORF42 and ORF43) are also present on the POA1180 genome, but whether the chromate resistance genes of this phage can be mobilized, is not known. The POA1180 ORF46 product is similar to sulfate permeases belonging to the CysZ family and may mediate the uptake of sulfate subsequently utilized for the synthesis of sulfur-containing compounds in the cell. Another gene that might be important for *Ochrobactrum* metabolism is ORF53 probably encoding a NAD(P) transhydrogenase. In addition, the right half of the POA1180 phage genome contains genes for an ATPase, transcriptional regulators, a partitioning protein, an exonuclease and the endolysin. However, for most genes located in this region, functional predictions could not be made.

**Figure 5:**
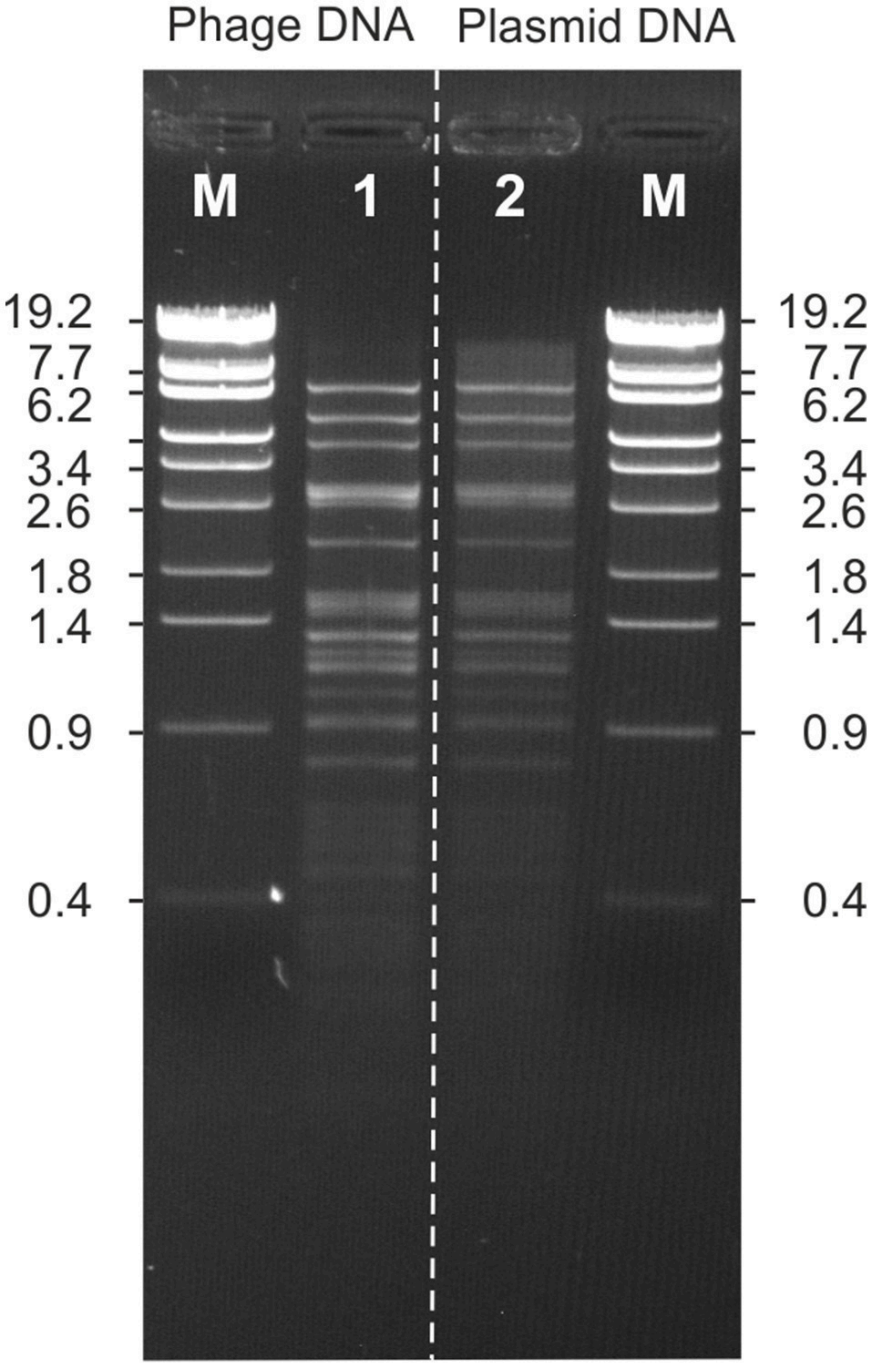
Comparison of the EcoRV restriction patterns of the POI1126 phage (1) and plasmid prophage (2).

Phage POI1126 possesses a genome of 60,065 bp with a GC-content of 56.2%. Eighty putative genes were assigned, which are equally located on both DNA strands (Figure 4). The function of 31 gene products could be predicted on the basis of similarities to known proteins. The closest related phages of POI1126 are the *S. meliloti* phage PCB5, *Erwinia pyrifoliae* phage PEp14 and the *Burkholderia cenocepacia* phages DC1, Bcep22, and BcepIL02. While the lifestyle and morphology of PCB5 have not been documented yet, the other phages are podoviruses like POI1126 (Gill et al., 2011; Lynch et al., 2012). The three *Burkholderia* phages were originally isolated from soil. It has still to be clearified whether these phages are virulent or temperate since they were unable to form stable lysogens. Although, a recombinase gene and an *attP* site have been detected on the phage genomes the phages may be unable to successfully integrate into the host chromosome. In contrast, phage POI1126 was isolated from an *O. intermedium* strain by induction with mitomycin C suggesting a temperate lifestyle. The analysis of the POI1126 genome disclosed that the phage is almost identical to an unnamed plasmid of the *O. intermedium* strain LMG 3301 (ACQA01000004). We only found one nucleotide variation (deletion) in ORF30. The frame shift mutation led to an exchange of seven C-terminal amino acids in the predicted DNA-methylase protein. The strong homologies to the plasmid of strain LMG 3301 inspired us to analyse the plasmid content of *O. intermedium* strain O1126. This strain indeed contains a plasmid that showed an identical EcoRV restriction pattern as phage POI1126 (Figure 5). From these data, it can be assumed that both *O. intermedium* strains, O1126 and LM3301, harbor a temperate phage whose prophage replicates as plasmid. The sequence of the LMG 3301 plasmid deposited at NCBI is framed by a terminal direct repeat of 389 bp. We analyzed the corresponding DNA regions of POI1126 and the plasmid prophage by PCR but did not detect this repetitive sequence. As a consequence the first and last ORF of the LMG 3301 sequence encoding putative DNA methylases are merged in strain O1126. The resulting methylase gene (ORF30) is by far the largest gene of POI1126 (14.526 bp) and preceded by a suitable ribosome binding site. The genome analysis of the phage revealed two ORFs (38 and 39) probably forming an operon that may be important for plasmid maintenance. Their products are similar to partitioning proteins. Other plasmid-associated genes could not be determined. On the other hand, a putative integrase gene (ORF65) was identified whose function is yet not known. The gene map of phage POI1126 shows that the left half of the genome mainly contains genes for structural proteins and virion assembly, while only few ORFs in the right half could be functionally assigned. It is possible that this part of the genome harbors other genes, which are required for plasmid replication.

**Figure 6:**
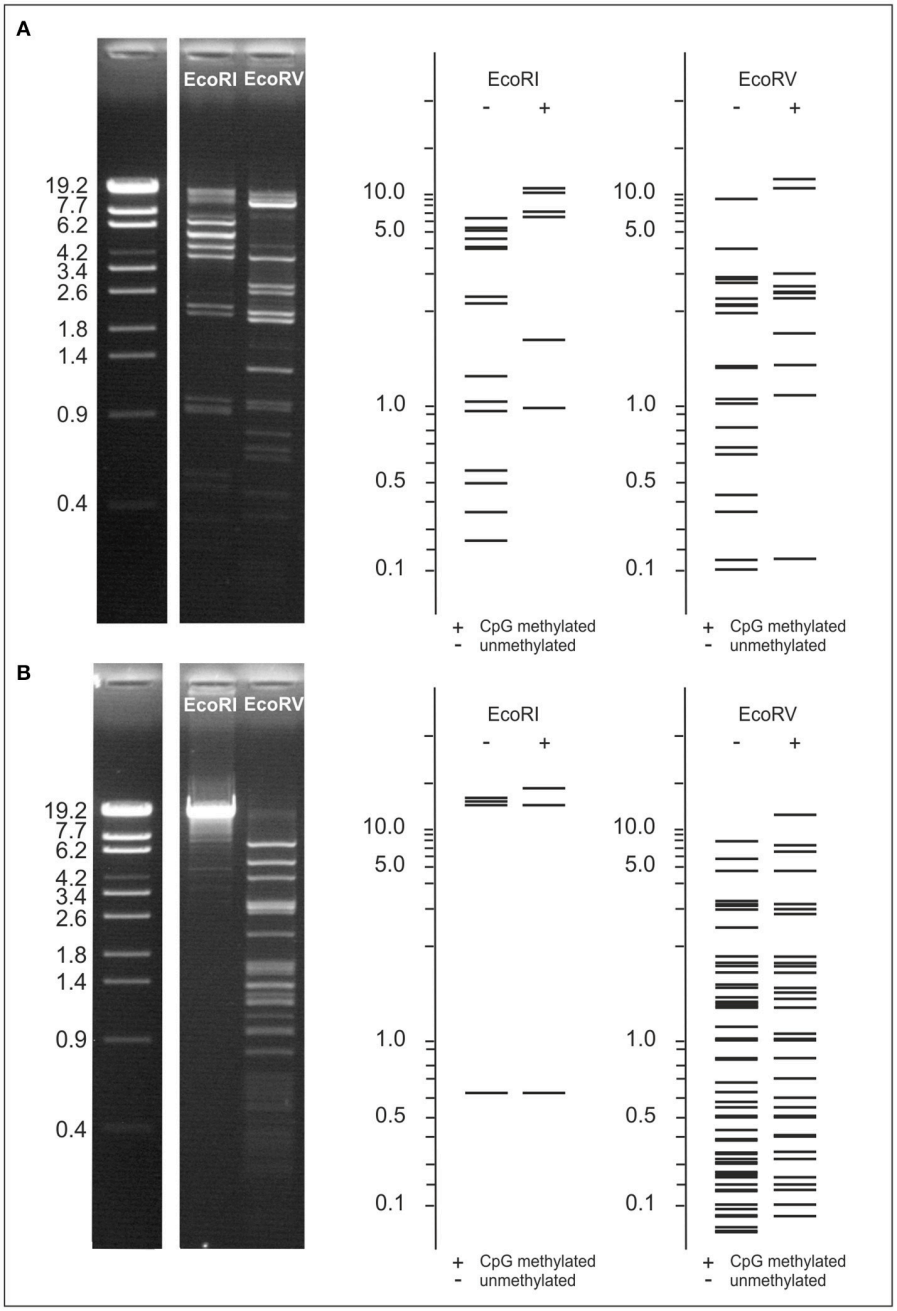
Examination of POA1180 and POI1126 DNA modifications. To compare restriction patterns of the phages, EcoRI and EcoRV digests of POA1180 **(A)** and POI1126 **(B)** were separated on 0.8% agarose gels. Sizes of restriction fragments were determined using the λ Eco130I reference marker. For both phages EcoRI and EcoRV restriction patterns were predicted by computer analysis to compare the *in silico* data with experimental results. Predicted restriction patterns are shown for unmethylated and CpG methylated DNA.

The phages POI1126 and POA1180 possess several genes encoding potential methyltransferases (Table S1). To find out whether the genomes of the phages are modified, their DNAs were digested with several restriction endonucleases. None of the obtained restriction patterns of POA1180 coincided with patterns determined by *in silico* analysis. Figure 6A presents EcoRI and EcoRV profiles of the POA1180 DNA. Using the NEB cutter software, restriction patterns of CpG methylated phage DNA were predicted but also these patterns did not agree with patterns obtained by digestion (Figure 6A). However, a superimposition of the computer-generated EcoRI profiles (but not of the EcoRV profiles) corresponded well with the restriction pattern observable in agarose gels. This suggests that the POA1180 phage DNA contains some modifications, which diverge from modifications known in *E. coli*. On the other hand, the EcoRI and EcoRV restriction patterns of phage POI1126 were in good agreement with patterns predicted for unmethylated DNA (Figure 6B). Thus, the DNA of this phage is either not modified or contains modifications which cannot be traced by the restriction endonucleases used.

## Discussion

In this study, temperate *Ochrobactrum* phages were isolated and characterized. Whereas, plasmids of *Ochrobactrum* have already been described (Chain et al., 2011) phages have yet not been investigated. Initial *in silico* analyses of published *Ochrobactrum* spp. genomes revealed that prophages are apparently widespread in this genus, similarly to the related genus *Brucella*, where numerous prophages have been identified (Hammerl et al., 2016). However, while to date only one active temperate phage could be isolated from *Brucella* spp., 72 out of 125 tested *Ochrobactrum* strains of different species released phage particles that caused plaques on indicator bacteria. The reason for this incongruency is yet not clear. It is conceivable that in *Brucella* spp. many prophages are defective. Compared to *Ochrobactrum*, the *Brucella* genome is much smaller. The genome reduction is associated with the adaption of brucellae to the intracellular lifestyle (Teyssier et al., 2004) and it is possible that prophage sequences are not conducive for the survival in the intra-host environment. Other reasons could be a high host specificity of *Brucella* phages or an immunity of *Brucella* strains to phage infection due to the presence of inherent prophages. Though, the close relationship between *Brucella* and *Ochrobactrum* does not really support this speculation. Many of the *Ochrobactrum* lysates revealed a rather broad lytic activity against non-lysogenic and lysogenic strains. In addition, as with *B. inopinata* phage BiPBO1, *Ochrobactrum* prophages could be readily induced by mitomycin C, UV, or heat treatment. Moreover, some *Ochrobactrum* strains released significant numbers of phage particles even under non-induced conditions (data not shown). Therefore, it remains obscure, why it is much easier to isolate temperate phages from *Ochrobactrum* than from *Brucella*.

Electron microscopy illustrated that temperate *Ochrobactrum* phages are morphologically diverse. Although, myoviruses are typically lytic while many siphoviruses can integrate into the host genome (Suttle, 2005), most *Ochrobactrum* lysates contained myoviruses. In some lysates two morphotypes (myovirus/podovirus and podovirus/siphovirus) were detected showing that these phages may coexist in the same cell. Phages belonging to different families generally share only little DNA similarity (Jarvis, 1984; Ackermann, 1992; Krylov et al., 1993; Wichels et al., 1998). The majority of the 19 isolated *Ochrobactrum* phages revealed clearly distinguishable restriction patterns. Some patterns were identical but in this case the respective phages varied in their host range indicating that they are related but not identical. Based on the obtained restriction patterns, genome sizes between 32 and 63 kb were calculated, a size range often found in phages (Hatfull, 2008). Southern hybridization experiments confirmed the diversity of the isolated phages. None of the phages revealed significant DNA homology to the myovirus POA1180. Podovirus POI1126 hybridized strongly to two other podoviruses (P1129 and P1187) but not to the remaining phages whereas *B. inopinata* siphovirus BiPBO1 showed similarity to P1129 and P1157. While the relationship of BiPB01 to the siphovirus P1157 was not surprising, we did not expect signals with P1157, because this phage had been classified as podovirus. A re-examination of the phage isolated from CsCl density-gradient band disclosed that the preparation contained both a podovirus and a siphovirus. The phages could obviously not be separated by CsCl density-gradient centrifugation. Since most *Ochrobactrum* strains are lysogenic or multilysogenic, prophages may be induced when infected with a phage from outside. As stated above some *Ochrobactrum* strains released high numbers of phage particles spontaneously during growth in culture media. Thus, there is always the possibility, that lysates are contaminated by endogenous phages and because of this fact, it is a challenge to find non-lysogenic, phage sensitive *Ochrobactrum* spp. strains to propagate phages that are of interest.

The analysis of the POA1180 phage genomes suggests that it is similarly organized like the genomes of many other temperate phages. The genome contains some functional gene modules, particularly for DNA packaging and virion assembly (Lima-Mendez et al., 2011). Though, for about half of the POA1180 genes, a functional assignment could not be made. One reason for this ambiguity could be that the closest relatives of POA1180 are prophages identified in other genera of the orders *Rhizobiales, Burkholderiales*, and *Rhodobacteriales* and not phage particles that have been characterized in detail. Nevertheless, typical for soil bacteria, some genes were identified on the POA1180 genome, which could be beneficial for its host. Resistance against chromate has been found in several bacteria including *Ochrobactrum* (Branco and Morais, 2013; Hora and Shetty, 2015), but to the best of our knowledge, the respective genes have yet not been identified on the genome of a temperate phage. The same holds true for the sulfate permease gene which may improve the supply of the host with sulfate (Pilsyk and Paszewski, 2009). These genes may give *Ochrobactrum* a selective advantage in the environment. Since phage POA1180 possesses a rather wide host range within the genus *Ochrobactrum* and because its DNA revealed some modification which may protect it from degradation, it is possible that other *Ochrobactrum* strains may benefit from lysogenic conversion. On the other hand, it becomes clear that those genes would not be beneficial for intracellular bacteria like *Brucella* and this may be one reason why active prophages are so rare in this genus. However, the isolation of phage BiPB01 from *B. inopinata* (Hammerl et al., 2016) and the occurrence of prophages in other non-classical *Brucella* species that are closely related to active *Ochrobactrum* phages (Scholz et al., 2016) suggest that those *Brucella* strains may be carrier of intact phages.

POI1126 is a temperate phage which replicates as plasmid during the lysogenic cycle. Whether POI1126 may integrate into the host chromosome has still to be investigated. The phage contains a putative integrase gene but it is possible that this gene is defective or that the attachment site *attP* is not functional or missing. The relationship of POI1126 to a plasmid of the *O. intermedium* strain LMG 3301 indicates that plasmid prophages might be common in this genus. Moreover, POI1126 is also related to podoviruses of other genera, e.g., *Burkholderia*. For *B. cenocepacia* phage Bcep22, whose genome showed a close relationship to the LMG 3301 plasmid as well, it has been reported that its prophage is unable to integrate into the host chromosome (Gill et al., 2011). Is it possible that this phage is a plasmid like POI1126? Further experiments are needed to elucidate the lifestyle of this group of podophages.

## Author contributions

JH, CJ, HS, KN, and SH designed the study. JH, CJ, and JR performed the experiments. JH, CJ, HS, JR, and SH analyzed the data. All authors prepared the tables and figures, wrote, and edited the manuscript.

### Conflict of interest statement

The authors declare that the research was conducted in the absence of any commercial or financial relationships that could be construed as a potential conflict of interest.
