# Supplementary material for: Prevalence, Host Range, and Comparative Genomic Analysis of Temperate Ochrobactrum Phages
Source: Front Microbiol. 2017 Jun 30;8:1207. doi: 10.3389/fmicb.2017.01207 (PMC5492332; doi:10.3389/fmicb.2017.01207)
Supplement: Supplementary file 2 [file Table2.DOCX]

Supplementary Material

Prevalence, host range and comparative genomic analysis of temperate *Ochrobactrum* phages

**Claudia Jäckel^†^, Stefan Hertwig^†^, Holger C. Scholz, Karsten Nöckler, Jochen Reetz, and Jens A. Hammerl^*^**

^†^ Both authors contributed equally to this work.

*** Correspondence:** Jens Andre Hammerl: [jens-andre.hammerl@bfr.bund.de](mailto:jens-andre.hammerl@bfr.bund.de)

# Supplementary Material Table S2

**(A) *Ochrobactrum anthropi* genomes**

| **Strain** | **Location** | **Related phage (Host organism)** | | | **Position** | | **Size (kb)** | **Predicted status** | | **Score** | **CDS** | **GC-content (%)** |
| --- | --- | --- | --- | --- | --- | --- | --- | --- | --- | --- | --- | --- |
| ***O. anthropi* ATCC 49188** | | | | | | | | | | | | |
| PP1 | CHR1 | 3 (*Rhizobium*) | | | 247,774-265,325 | | 17.5 | Incomplete | | 60 | 22 | 55.6 |
| PP2 | CHR1 | RE-2010 (*Salmonella*) | | | 1,574,194-1,591,469 | | 17.2 | Intact | | 100 | 17 | 53.7 |
| PP3 | CHR1 | S1 (*Stenotrophomonas*) | | | 1,869,085-1,888,159 | | 19.0 | Intact | | 100 | 20 | 52.9 |
| PP4 | CHR1 | phiE202 (*Burkholderia*) | | | 2,410,980-2,433,280 | | 22.3 | Intact | | 100 | 25 | 55.8 |
| PP5 | CHR2 | ENT39118 (*Cronobacter*) | | | 493,235-515,845 | | 22.6 | Incomplete | | 20 | 13 | 65.8 |
| ***O. anthropi* ATCC 49687 (OAB)** | | | | | | | | | | | | |
| PP1 | CHR1 | 1/44 (*Shewanella*) | | | 1,328,210-1,346,235 | | 18.0 | Questionable | | 80 | 21 | 52.8 |
| PP2 | CHR2 | vB_BanS_Tsamsa (*Bacillus*) | | | 741,545-754,592 | | 13.0 | Incomplete | | 20 | 13 | 65.9 |
| ***O. anthropi* ML7** | | | | | | | | | | | | |
| PP1 | CHR | RC1 (*Rhodobacter*) | | | 1,565,908-1,609,364 | | 43.4 | Intact | | 100 | 46 | 57.5 |
| PP2 | CHR | AmM-1 (*Aurantimonas*) | | | 2,233,077-2,289,880 | | 56.8 | Intact | | 150 | 63 | 56.8 |
| PP3 | CHR | BP-4795 (*Enterobacteria*) | | | 2,312,791-2,325,305 | | 12.5 | Questionable | | 70 | 17 | 55.7 |
| PP4 | CHR | ST64B (*Salmonella*) | | | 2,407,623-2,425,470 | | 17.8 | Questionable | | 80 | 15 | 54.2 |
| PP5 | CHR | 16-3 (*Rhizobium*) | | | 4,163,402-4,204,448 | | 41.0 | Intact | | 110 | 52 | 55.5 |
| PP6 | CHR | G (*Bacillus*) | | | 4,722,661-4,738,667 | | 16.0 | Questionable | | 70 | 16 | 54.8 |
| ***O. anthropi* W13P3** | | | | | | | | | | | | |
| PP1 | CHR | RcapMu (*Rhodobacter*) | | | 261,187-293,755 | | 32.5 | Questionable | | 90 | 37 | 57.3 |
| PP2 | CHR | RR1-A (*Rhizobium*) | | | 1,257,777-1,304,080 | | 46.3 | Questionable | | 80 | 39 | 57.0 |
| PP3 | CHR | phiRSA1 (*Ralstonia*) | | | 1,298,428-1,313,799 | | 15.3 | Questionable | | 70 | 14 | 54.2 |
| PP4 | CHR | BP-4795 (*Enterobacteria*) | | | 1,938,472-1,961,127 | | 22.6 | Questionable | | 80 | 33 | 54.7 |
| PP5 | CHR | UFV-P2 (*Pseudomonas*) | | | 3,786,190-3,822,637 | | 36.4 | Questionable | | 70 | 22 | 49.7 |
| PP6 | CHR | PBC5 (*Sinorhizobium*) | | | 3,815,404-3,822,541 | | 7.1 | Incomplete | | 50 | 5 | 52.3 |
| ***O. anthropi* 60a** | | | | | | | | | | | | |
| PP1 | CHR | vB_RglS_P106B (*Rhizobium*) | | | 515,639-526,157 | | 10.5 | Incomplete | | 60 | 14 | 55.1 |
| PP2 | CHR | phiEaH2 (*Erwinia*) | | | 763,975-772,600 | | 8.6 | Incomplete | | 40 | 9 | 56.3 |
| ***O. anthropi* CTS-325** | | | | | | | | | | | | |
| PP1 | CHR | RHEph06 (*Rhizobium*) | | | 2,964,938-2,976,708 | | 11.7 | Questionable | | 80 | 16 | 55.6 |
| **PP2** | CHR | PAU (*Sphingomonas*) | | | 4,410,643-4,433,347 | | 22.7 | Questionable | | 80 | 23 | 50.8 |
| ***O. anthropi* FRAF13** | | | | | | | | | | | | |
| PP1 | CHR | | PaV_LD (*Planktothrix*) | 1,305,436-1,314,025 | | 8.5 | | | Incomplete | 60 | 11 | 59.6 |
| PP2 | CHR | | AmM-1 (*Aurantimonas*) | 1,401,494-1,426,811 | | 25.3 | | | Intact | 150 | 27 | 57.3 |
| PP3 | CHR | | F116 (*Pseudomonas*) | 1,428,610-1,454,793 | | 26.1 | | | Incomplete | 30 | 17 | 55.9 |
| PP4 | CHR | | 16-3 (*Rhizobium*) | 3,391,347-3,445,398 | | 54.0 | | | Questionable | 90 | 75 | 54.8 |

**(B) *Ochrobactrum intermedium* genomes**

| **Strain** | **Location** | | **Related phage (Host organism)** | | **Position** | | **Size (kb)** | | **Predicted status** | | **Score** | **CDS** | **GC-content (%)** |
| --- | --- | --- | --- | --- | --- | --- | --- | --- | --- | --- | --- | --- | --- |
| ***O. intermedium* LMG 3301** | | | | | | | | | | | | | |
| PP1 | CHR1 | RC1 (*Rhodobacter*) | | 1,796,867-1,837,707 | | 48.8 | | | Intact | | 150 | 53 | 58.2 |
| PP2 | CHR1 | 3 (*Rhizobium*) | | 3,660,834-3,694,018 | | 33.1 | | | Questionable | | 90 | 20 | 55.1 |
| PP3 | CHR1 | PBC5 (*Sinorhizobium*) | | 4,697,905-4,725,392 | | 27.4 | | | Incomplete | | 40 | 31 | 56.9 |
| PP4 | CHR2 | 12B12 (*Vibrio*) | | 1,796,867-1,837,707 | | 40.8 | | | Intact | | 150 | 53 | 58.2 |
| **O. intermedium 2745-2** | | | | | | | | | | | | | |
| PP1 | CHR | AmM-1 (*Aurantimonas*) | | 675,501-726,589 | | 51.0 | | | | Intact | 150 | 64 | 57.3 |
| PP2 | CHR | PBC5 (*Sinorhizobium*) | | 1,425,012-1,460,347 | | 35.3 | | | | Questionable | 90 | 32 | 56.4 |
| PP3 | CHR | 16-3 (*Rhizobium*) | | 2,799,698-2,839,799 | | 40.1 | | | | Questionable | 90 | 54 | 56.4 |
| PP4 | CHR | vB_RglS_P106B (*Rhizobium*) | | 3,512,929-3,528,432 | | 15.5 | | | | Questionable | 70 | 20 | 55.4 |
| ***O. intermedium* M86** | | | | | | | | | | | | | |
| PP1 | CHR | vB_EcoM_ECO1230_10 (*Escherichia*) | | 258,398-270,698 | | 12.3 | | | Incomplete | | 60 | 18 | 57.7 |
| PP2 | CHR | AmM-1 (*Aurantimonas*) | | 997,490-1,051,835 | | 54.3 | | | Intact | | 150 | 57 | 56.7 |
| PP3 | CHR | 12B12 (*Vibrio*) | | 2,278,388-2,308,901 | | 30.5 | | | Intact | | 130 | 44 | 57.7 |
| PP4 | CHR | 16-3 (*Rhizobium*) | | 2,935,040-2,952,367 | | 17.3 | | | Incomplete | | 10 | 23 | 56.2 |
| PP5 | CHR | ep3 (*Escherichia*) | | 3,654,897-3,678,421 | | 23.5 | | | Intact | | 150 | 30 | 57.5 |
| PP6 | CHR | AmM-1 (*Aurantimonas*) | | 4,544,888-4,559,280 | | 14.3 | | | Questionable | | 70 | 25 | 56.6 |
| PP7 | CHR | RHEph06 (*Rhizobium*) | | 4,567,687-4,581,218 | | 13.5 | | | Questionable | | 80 | 18 | 56.3 |
| PP8 | CHR | RR1-A (*Rhizobium*) | | 5,107,821-5,138,359 | | 30.5 | | | Questionable | | 80 | 9 | 58.3 |
| PP9 | CHR | MaMV-DC (*Micrococcus*) | | 5,131,937-5,138,881 | | 6.9 | | | Incomplete | | 30 | 14 | 62.5 |
| PP10 | CHR | Virus (*Phaeoceros*) | | 5,164,144-5,173,437 | | 9.2 | | | Questionable | | 80 | 9 | 59.3 |
| ***O. intermedium* CCUG 57381 (299E)** | | | | | | | | | | | | | |
| PP1 | CHR | W.Ph. (*Bacillus*) | | 467,372-478,017 | | 10.6 | | | Incomplete | | 10 | 20 | 57.9 |
| PP2 | CHR | Chlorella virus (*Acanthocystis*) | | 554,239-562,539 | | 8.3 | | | Incomplete | | 10 | 10 | 58.6 |
| ***O. intermedium* 2745-2** | | | | | | | | | | | | | |
| PP1 | CHR | AmM-1 (*Aurantimonas*) | | 675,501-726,589 | | | | 51 | Intact | | 150 | 64 | 57.3 |
| *O. intermedium* KCJK1738 | | | | | | | | | | | | | |
| PP1 | CHR | PaV_LD (*Planktothrix*) | | 1,305,436-1,314,025 | | | | 8.5 | Incomplete | | 60 | 11 | 59.6 |
| PP2 | CHR | AmM-1 (*Aurantimonas*) | | 1,401,494-1,426,811 | | | | 25.3 | Intact | | 150 | 27 | 57.3 |
| PP3 | CHR | F116 (*Pseudomonas*) | | 1,428,610-1,454,793 | | | | 26.1 | Incomplete | | 30 | 17 | 55.9 |
| PP4 | CHR | 16-3 (*Rhizobium*) | | 3,391,347-3,445,398 | | | | 54.0 | Questionable | | 90 | 75 | 54.8 |
| ***O. intermedium* T** | | | | | | | | | | | | | |
| PP1 | CHR | 16-3 (*Rhizobium*) | | 1,514,235-1,532,543 | | | | 18.3 | Incomplete | | 10 | 21 | 55.1 |
| PP2 | CHR | phiLM21 (*Sinorhizobium*) | | 3,241,539-3,256,136 | | | | 14.5 | Questionable | | 90 | 19 | 55.4 |

**(C) *Ochrobactrum* spp. genomes**

| **Strain** | **Location** | **Related phage (Host organism)** | | **Position** | **Size (kb)** | **Predicted status** | **Score** | **CDS** | **GC-content (%)** |
| --- | --- | --- | --- | --- | --- | --- | --- | --- | --- |
| ***O. pseudogrignonense* K8** | | | | | | | | | |
| PP1 | CHR1 | vB_PmaS_IMEP1 (*Paracoccus*) | 539,520-553,235 | | 13.7 | Intact | 120 | 17 | 56.3 |
| PP2 | CHR1 | 16-3 (*Rhizobium*) | 3,306,195-3,343,193 | | 36.9 | Questionable | 90 | 30 | 51.4 |
| PP3 | CHR2 | Tripp (*Paenibacillus*) | 83,828-96,979 | | 13.1 | Intact | 10 | 19 | 56.6 |
| ***Ochrobactrum* sp. CDB2** | | | | | | | | | |
| PP1 | CHR | vB_PmaS_IMEP1 (*Paracoccus*) | | 928,215-942,041 | 13.8 | Intact | 150 | 20 | 56.0 |
| PP2 | CHR | ep3 (*Escherichia*) | | 2,876,924-2,903,397 | 26.4 | Incomplete | 20 | 22 | 51.5 |
| PP3 | CHR | 16-3 (*Rhizobium*) | | 3,320,785-3,382,706 | 61.9 | Intact | 110 | 77 | 52.1 |
| ***O. rhizosphaerae* SJY1** | | | | | | | | | |
| PP1 | CHR | RC1 (*Rhodobacter*) | | 1,335,640-1,376,599 | 40.9 | Intact | 150 | 56 | 54.0 |
| PP2 | CHR | vB_PmaS_IMEP1 (*Paracooccus*) | | 1,868,599-1,882,507 | 13.9 | Intact | 120 | 18 | 55.0 |
| PP3 | CHR | ep3 (*Escherichia*) | | 2,768,075-2,783,204 | 15.1 | Incomplete | 50 | 22 | 52.3 |
| PP4 | CHR | 16-3 (*Rhizobium*) | | 3,535,823-3,627,705 | 91.8 | Intact | 150 | 112 | 52.9 |
| PP5 | CHR | AmM-1 (*Aurantimonas*) | | 4,304,854-4,345,382 | 40.5 | Intact | 150 | 58 | 52.8 |
| PP6 | CHR | AmM-1 (*Aurantimonas*) | | 4,914,810-4,946,994 | 32.1 | Intact | 150 | 40 | 55.0 |
| PP7 | CHR | 16-3 (*Rhizobium*) | | 5,191,258-5,232,450 | 41.1 | Intact | 110 | 49 | 51.6 |
| ***Ochrobactrum* sp. UNC390CL2Tsu3S39 BS36** | | | | | | | | | |
| PP1 | CHR | CR44b (*Citrobacter*) | | 17,743-27,943 | 10.2 | Incomplete | 20 | 16 | 50.8 |
| PP2 | CHR | Tb (*Brucella*) | | 23,087-49,664 | 26.5 | Incomplete | 20 | 35 | 53.9 |
| PP3 | CHR | Cd (*Azospirillum*) | | 64,279-87,408 | 23.1 | Intact | 100 | 30 | 55.4 |

| ***Ochrobactrum* sp. EGD-AQ16** |
| --- |

| PP1 | CHR | Stx2 converting phage I (*Escherichia*) | 8,229-21,994 | 13.7 | Incomplete | 20 | 15 | 53.2 |
| --- | --- | --- | --- | --- | --- | --- | --- | --- |
| PP2 | CHR | RSL2 (*Ralstonia*) | 2,584,592-2,592,106 | 7.5 | Incomplete | 40 | 9 | 57.6 |
| PP3 | CHR | Chlorella virus FR483 (Paramecium) | 2,652,377-2,667,343 | 14.9 | Incomplete | 10 | 17 | 58.0 |

Abbreviations: WGS, whole genome sequence; data request: 15 June 2016
